# The Effects of Co-Treatment of 9-*cis*-Retinoic Acid and 15-Deoxy-Δ (12,14)-prostaglandin J2 on Microglial Activation

**DOI:** 10.3390/molecules16054045

**Published:** 2011-05-17

**Authors:** Pei-Chien Hsu, Huey-Jen Tsay, Thomas J. Montine, Feng-Shiun Shie

**Affiliations:** 1 Institute of Neuroscience, National Yang-Ming University, Taipei, Taiwan; E-Mails: pachann99@yahoo.com.tw (P.-C.H.); hjtsay@ym.edu.tw (J.-J.T.); 2 Department of Pathology, University of Washington, Harborview Medical Center, Seattle, WA 98223, USA; E-Mail: tmontine@u.washington.edu; 3 Division of Mental Health and Addiction Medicine, the Institute of Population Health Sciences, National Health Research Institutes, 35 Keyan Road, Zhunan, Miaoli County 350, Taiwan

**Keywords:** Alzheimer’s disease, Aβ, microglia, PPARγ, neuroinflammation, pro-NGF

## Abstract

Microglial activation plays an important role in the regulation of neuronal function and contributes to the development of neurodegeneration in Alzheimer’s disease (AD). Activation of nuclear peroxisome proliferator-activated receptor gamma (PPARγ) by an endogenous agonist, 15-deoxy-Δ(12,14)-prostaglandin J2 (15d-PGJ2), has been shown to be beneficial in many diseases with aberrant immune responses. Here, we report that co-treatment with 15d-PGJ2 and its synergistic partner, 9-*cis*-retinoic acid (RA), may modulate, but not abolish, microglial immune response activated by β-amyloid (Aβ) and interferon gamma (IFNγ). The co-treatment of RA and 15d-PGJ2 inhibited Aβ/IFNγ-activated immune response in primary microglia, as evidenced by suppressed expression of inducible nitric oxide synthase (iNOS) and cyclooxygenase-2 (COX-2); and the effect was not affected by treatment with a PPARγ antagonist, GW9662. Data suggest that PPARγ activation may not contribute to the anti-inflammatory properties of the co-treatment. The co-treatment promoted microglial Aβ clearance in cultures; and the effect can be prevented by blocking PPARγ activation using GW9662. The effects of the co-treatment on Aβ clearance may be PPARγ-dependent. Intriguingly, secretion of microglial pro-nerve growth factor (pro-NGF) was inhibited by Aβ/IFNγ treatment in a dose-dependent manner, suggesting that secretion of microglial pro-NGF may not contribute to the Aβ/IFNγ-activated microglial immune response. Taken together, the co-treatment may be beneficial for AD therapy; however, our data suggest that multiple mechanisms may underlie the beneficial effects of the co-treatment and are not limited to PPARγ activation only.

## 1. Introduction 

Alzheimer’s disease (AD) is responsible for the majority of dementia cases in the elderly. Although the etiology of AD remains controversial, microglia are thought to play an important role in the pathogenesis of AD. In the diseased brain, microglia become activated and produce various effectors that are critical for neuronal survival during pathological events [1,2,3,4]. These effectors include cytokines, chemokines, reactive oxygen and nitrogen species, prostaglandins (PGs), and neurotrophic factors [5,6,7]. Microglial activation can be neuroprotective by phagocytosing β-amyloid (Aβ), the major component of senile plaques in AD brain, and by releasing neurotrophic factors, such as neurotrophins, to promote neuronal survival. Indeed, Aβ is one of a handful of endogenous agents known to activate microglial innate immunity and has been used for microglial activation as a model in AD research; however, the forms and sizes of Aβ responsible for triggering microglia-mediated inflammation remain elusive [8,9,10]. Microglial activation also accelerates oxidative stress by inducing pro-inflammatory proteins and cytokines as well as reactive oxygen and nitrogen species, which in turn can exacerbate AD pathogenesis. Thus, there is an emerging consensus that discovering means to promote the beneficial functions of microglial activation while diminishing the detrimental effects could be an effective therapeutic approach to AD.

Due to its anti-inflammatory properties, peroxisome proliferator-activated receptor gamma (PPARγ) has recently received increasing attention in the field of AD research [11]. PPARγ and two other genetically distinct isoforms (PPARα and PPARβ/δ) are members of a nuclear hormone receptor superfamily that are intimately involved in various regulations of gene expressions that include glucose and lipid metabolism, cell differentiation, apoptosis, inflammation, and carcinogenesis [12,13,14]. It appears that transcription activity of PPARs is synergistically enhanced by heterodimer formation with retinoid X receptors (RXRα, RXRβ, RXRγ) that bind specific peroxisome proliferators response elements (PPREs) in target genes [15,16]. Synergistic activation by PPAR-RXR heterodimer may affect various protein functions involved in anti-inflammation. The facts that PPARγ can be activated by subsets of nonsteroidal anti-inflammatory drugs (NSAIDs) support the notion that PPARγ might have potential in modulating microglial activation in neurodegenerative diseases [17,18,19,20].

PPARγ and RXRs are widely expressed in a variety of tissues [21]. In the central nervous system (CNS), the presence of receptors was observed in both neurons and glia [22]. Ligands of PPARγ include anti-diabetic drugs (such as thiazolidinediones and pioglitazone), polyunsaturated fatty acids, some high-affinity tyrosine derivatives, fibrates, and the naturally occurring prostaglandin metabolite 15-deoxy-Δ(12,14)-prostaglandin J2 (15-d PGJ2). For RXRs, 9-*cis*-retinoic acid (RA) binds all isoforms and synergistically activate PPARγ with PPARγ ligands [23,24]. The anti-inflammatory effects of PPARγ agonists on monocyte/macrophage activation were first reported by Ricote *et al.* [25] and by Jiang *et al.* [26]. It was found that PPARγ agonists, such as troglitazone, 15d-PGJ2, and a synthetic PPARγ agonist, BRL49653, may attenuate interferon-γ (IFNγ)-stimulated activation of monocytes/macrophages as shown in inducible nitric oxide synthase (iNOS) and matrix metalloprotease-9 (MMP-9). PPARγ agonists also inhibit phorbol myristyl acetate (PMA)-induced pro-inflammatory cytokines, such as TNFα. The effects of PPARγ activation on microglial activation were also reported [27,28,29]. Although they came to different mechanistic conclusions, the studies agreed that PPARγ agonists are capable of suppressing lipopolysaccharide (LPS)-induced expressions of iNOS, cyclooxygenase 2 (COX-2), and pro-inflammatory cytokines. However, the evidence of a direct role of microglial pro-inflammatory proteins and cytokines in neurodegenerative diseases remains controversial [30,31].

The pro-form of nerve growth factor (pro-NGF) has been implicated in microglia-induced apoptosis in developing eyes [32]. Pro-NGF, a high molecular weight precursor of NGF, is secreted as well as cleaved into the mature form of NGF for secretion by the endoprotease furin within the trans-Golgi network [33 for review]. NGF and pro-NGF are ligands for two known receptors, tropomyosin-related kinase (TrkA) receptor tyrosine kinase and a pan-neurotrophin receptor (p75NTR). Whereas the TrkA receptor is a member of a family of receptor tyrosine kinases, p75NTR belongs to the Fas/tumor necrosis factor receptor (TNFR) family, which is known for its functions in apoptosis. With high affinity to p75NTR, pro-NGF induces p75NTR-dependent apoptosis, while activating TrkA receptor, mainly via NGF binding, is thought to promote neuronal survival. Others have speculated that up-regulation of pro-NGF in diseased regions of AD brain suggests a pro-apoptotic role of pro-NGF in AD [34,35]. Of note, pro-NGF is the major form of NGF in both normal and diseased brains. It remains unclear whether pro-NGF is involved in Aβ-induced neurotoxicity [36] and whether Aβ deposition contributes to the changes of pro-NGF levels. The role of secreted neurotrophin from microglia in AD pathogenesis also is unclear. In this study, we used the co-treatment of ligands for RXR and PPARγ to maximize the activation of PPARγ and to investigate the effects of the co-treatment of RA and 15d-PGJ2 in primary microglia on Aβ-induced immune activation, Aβ clearance, and secretion of pro-NGF.

## 2. Results and Discussion 

### 2.1. Aβ/IFNγ-induced microglial immune response was suppressed by the co-treatment of RA and 15d-PGJ2

Activation of PPARγ was achieved by co-treatment of RA and 15d-PGJ2, while immune response in microglia was measured by the inductions of inflammatory proteins COX-2 and iNOS. To achieve microglial activation at neurotoxicity levels, 15 μM of pre-aggregated Aβ1-42 was used in combination of IFNγ at a concentration of 10 ng/mL, as described elsewhere [37]. In this assay, Aβ was present in a mixture of monomer, polymers, and insoluble forms as identified by Western blot (data not shown). Thus, the Aβ-induced microglial activation in this study refers to the stimulation by a mixture of different forms and sizes of Aβ1-42. Primary mouse microglial cultures were pretreated with RA (10 nM) and 15d-PGJ2 (1 μM) followed by incubation of Aβ/IFNγ in the presence (10 μM for each) or in the absence of the inhibitor. Cell lysates were subjected to Western blot analysis for expressions of iNOS and COX-2 as an index of immune activation. Data were presented as % of the expression in cells treated with Aβ/IFNγ. Our results show that the co-treatment of RA and 15d-PGJ2 significantly reduced Aβ-induced expressions of COX-2 and iNOS in primary microglia (Table 1). Since 15d-PGJ2 is a natural ligand known for both prostaglandin D receptor 2 (DP2) and PPARγ, antagonists were used to test whether the corresponding pathways are involved. Results show that the induction of microglial COX-2 and iNOS was not affected by pretreatments of antagonists for PPARγ (GW9662) or DP2 (Bay-u3405, BAY), suggesting that PPARγ activation and DP2 signaling may not be involved. Similarly, bisindolylmaleimide (BIM), a PKC inhibitor, did not affect the effect of the co-treatment on expression of iNOS and COX-2. Thus, the co-treatment effect on anti-inflammation may be independent of PPARγ activation.

### 2.2. Co-treatment of RA and 15d-PGJ2 enhanced microglial Aβ clearance

**Figure 1:**
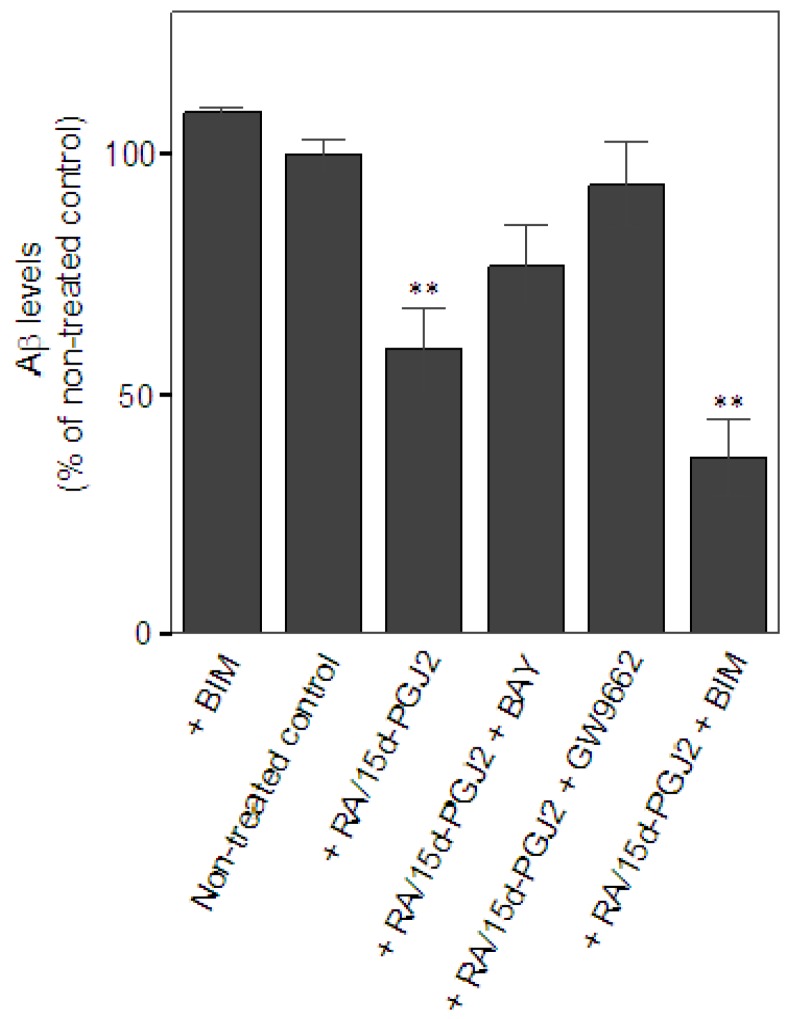
Effects of the co-treatment of RA and 15d-PGJ2 on Aβ clearance *in vitro*. Significance for comparisons with the non-treated controls was indicated as *p < 0.05, **p < 0.01.

Next, we examined the effect of the co-treatment of RA and 15d-PGJ2 on the capacity of microglia in Aβ clearance. The extent of microglial Aβ clearance was evaluated by measuring Aβ levels remaining in the cultural medium from primary microglial cultures after 24 h incubation with exogenous addition of Aβ as described in Experimental section. Aβ levels were quantified by the intensity of corresponding bands for Aβ monomer, approximately at 4 kDa, on Western blot. Data were presented as % of Aβ levels remaining in the cultures of non-treated controls. Treatment only with a PKC inhibitor, BIM, known to minimize microglial Aβ uptake [37] was serving as a negative control for Aβ clearance *in vitro*. At least four independent experiments were performed in each condition. Results showed that there was a significant reduction of Aβ in the medium when microglia were pretreated with RA and 15d-PGJ2 (59.3 ± 19.1% of non-treated control, *p* < 0.01) as compared to the non-treated controls (100 ± 6.3%), suggesting that the co-treatment promoted microglia-mediated Aβ clearance (Figure 1A). Data also showed that effect of the co-treatment on Aβ clearance was prevented by the treatment of the PPARγ antagonist, GW9662, (93.9 ± 17.5% of non-treated control). The DP2 antagonist, BAY, also reversed the effect of the co-treatment, but to a lesser extent (76.7 ± 17.7% of non-treated control). Intriguingly, we found that inhibition of PKC signaling using BIM did not alter the co-treatment’s effect on Aβ clearance (36.8 ± 16.5% of non-treated control). BIM alone did not significantly retard Aβ clearance (108.8 ± 2.9% of non-treated control). Because the treatment of BIM was known to inhibit Aβ uptake in microglia, these data suggest that the phagocytic pathway may not contribute to the co-treatment effect on Aβ clearance in our model. Investigation of the role of extracellular Aβ degradation in Aβ clearance will be important to understand the underlying mechanism of the beneficial effect of the co-treatment. Unlike the anti-inflammatory effect, the co-treatment of RA and 15d-PGJ2 in the enhancement of microglial Aβ clearance may be involved in PPARγ activation and DP2 signaling. This notion supports our previous hypothesis that the anti-inflammatory properties and phagocytic activity during microglial activation are under control of distinct regulatory pathways, which is in agreement with findings from others [38].

### 2.3. Aβ treatment inhibited the secretion of microglial pro-NGF independent of the status of microglial activation

**Figure 2:**
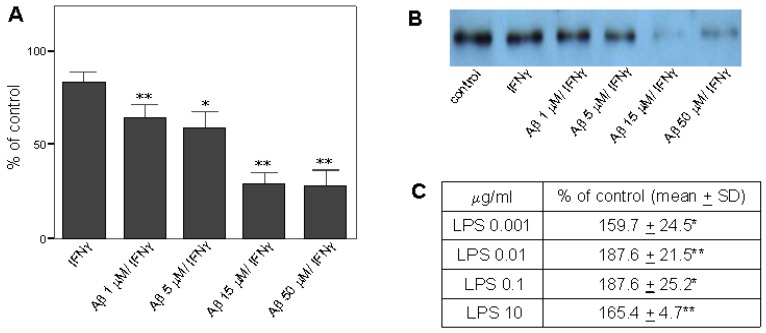
The effect of the treatment of Aβ/IFNγ or LPS on the secretion of pro-NGF in primary microglia. **(a)** Reduced secretion of microglial pro-NGF in a dose-dependent manner by Aβ/IFNγ treatment. **(b)** Representative Western blot of the secretion of microglial pro-NGF by Aβ/IFNγ treatment. **(c)** Elevated secretion of microglial pro-NGF by LPS treatment. Significance for comparisons with the controls was indicated as *p < 0.05, **p < 0.01, n ≥ 3.

Recently, pro-NGF has been reported to be pro-apoptotic and may be involved in various pathological events [32,39]. Others have reported that pro-NGF is increased in AD entorhinal cortex and it may be associated with the learning impairment in mice [34,35,40]; however, the cellular source of increased pro-NGF in diseased regions of AD brain is unreported. To correlate the level of microglial pro-NGF to microglial immune response, we examined the secretion and the intracellular level of pro-NGF in primary microglia. The levels of secreted pro-NGF in primary microglial cultures activated by Aβ/INFγ or by LPS were evaluated by Western blot as described in the Experimental section. Data were presented as % of basal secreted levels in non-treated controls. Data showed that pro-NGF was found both in the cell lysate and the culture medium from primary microglial cultures. Intracellular level of pro-NGF remained unchanged regardless of any experimental treatments (data not shown). Surprisingly, the secretion of microglial pro-NGF was reduced in a dose-dependent manner by Aβ/IFNγ treatment; however, IFNγ *per se* may not be the major contributing factor because IFNγ alone did not significantly reduce the secretion of microglial pro-NGF (Figures 2A, B). Levels of pro-NGF in % of the controls for IFNγ alone, 1 μM of Aβ plus IFNγ, 5 μM of Aβ plus IFNγ, 15 μM of Aβ plus IFNγ, and 50 μM of Aβ plus IFNγ were 83.2 ± 10.4 (ns), 64.4 ± 12.7 (*p* < 0.01), 59.0 ± 15.5 (*p* < 0.05), 29.4 ± 18.3 (*p* < 0.01), and 28.0 ± 14.9 (*p* < 0.01), respectively. The maximal reduction of secreted pro-NGF occurred when cells were treated with 15 μM or more of Aβ, a dose that elevated expression of COX-2 and iNOS in primary microglia. In contrast, LPS exposure, ranging from 0.001 to 10 μg/mL, significantly elevated secretion of microglial pro-NGF by, at least, 60% of the controls (Figure 2C). Data suggest that the secretion of microglial pro-NGF was regulated differentially by Aβ and LPS and may be independent of the status of microglial activation. The co-treatment of RA and 15d-PGJ2 did not alter pro-NGF secretion from microglia in both basal (109.2 ± 11.6% of the controls) and Aβ/IFNγ-stimulated conditions (37.1 ± 19.9% of the controls) as compared to the controls and Aβ/IFNγ-activated microglia (29.4 ± 18.3% of the controls), respectively. Interestingly, the level of pro-NGF was reduced from the basal conditions by a PKC inhibitor, BIM (63.0 ± 20.9). As compared to Aβ/IFNγ-activated microglia, BIM did not show significant changes on pro-NGF level in the treatment combined with Aβ (25.5 ± 11.6% of the control) or with Aβ plus the co-treatment (33.9 ± 26.3% of the control). The data indicate that the secretion of pro-NGF from microglia was not positively correlated with microglial immune response and may not have contributed to Aβ-activated microglia-mediated neuroinflammation. We speculate that secretion of microglial pro-NGF may have implications for microglial function beyond its pro-apoptotic property. To examine whether cell viability contribute to the effect of Aβ/IFNγ on the reduction of secreted pro-NGF, MTT assay was performed. Data indicated that Aβ at level of 15 μM in combination of IFNγ treatment for 24 h did not cause detectable cell toxicity in treated primary microglia (99.8 ± 35.9% of controls). MTT assay showed that the co-treatment of RA and 15d-PGJ2 did reduce cell viability (55.8 ± 13.2% of control) and the reduction of cell viability was not changed by the treatment with GW9662 (59.9 ± 24.6% of control) or by BAY (48.0 ± 11.8% of control). However, we did not observe any indications of reduction of cell numbers in cultures. Thus, cell viability may not be a contributing factor to the reduced secretion of pro-NGF in primary microglia.

**Figure 3:**
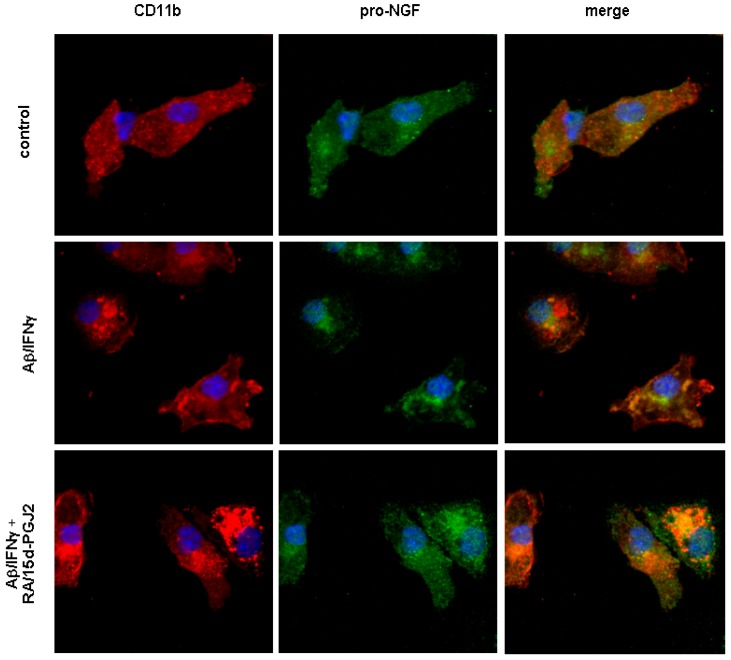
Immunoreactivity of intracellular pro-NGF in primary microglia.

To visualize intracellular localization of pro-NGF, confocal microscopy using antibodies against pro-NGF or CD11b, a marker for activated microglia, was applied. Our data revealed the presence of pro-NGF immunoreactivity in mouse primary microglia (Figure 3). In controls, pro-NGF immunoreactivity was found in the entire cell body with a partial co-localization between pro-NGF immunoreactivity and a microglial marker, CD11b. Activation of microglia by the treatment of 15 μM of Aβ along with IFNγ enhanced co-localization between CD11b and pro-NGF, while the peri-nuclear accumulation of pro-NGF immunoreactivity became apparent, which may have contributed to the reduced secretion of pro-NGF. The co-treatment of RA and 15d-PGJ2 appears to have further enhanced the co-localization between CD11b and pro-NGF where the pro-NGF immunoreactivity was sequestered in larger aggregates, mostly co-localized with CD11b positive vehicles. Of note, the co-treatment showed an increased immunoreactivity of CD11b in microglia as compared to those of cells with or without Aβ/IFNγ treatment.

Microglial activation is a double-edged sword. It can be both detrimental and beneficial; microglia may exacerbate neuroinflammation through autocrine/paracrine effects while promoting neuronal survival through phagocytic activity and by release of neurotrophic factors. Thus, a favorable combination of diminished microglia-mediated neuroinflammation and enhanced Aβ clearance may be critical in AD therapy. Here, we showed that the co-treatment of 15d-PGJ2 along with RA exerted a beneficial effect through distinct signaling pathways on Aβ-activated microglia evidenced by enhanced Aβ clearance and reduced immune activation, as measured by reductions of COX-2 and iNOS expression in primary microglia. Our data support the notion that the co-treatment may be beneficial in AD therapy. However, the mechanisms underlying the protective effects of the co-treatment are involved in multiple targeting and are not limited to activation of PPARγ. Since elevated expression of COX-2 and iNOS may lead to generation of many down-stream pro-inflammatory mediators (such as cytokines, prostaglandins, and nitric oxide derivatives) acting through various receptor-coupled signaling pathways [27,31,37], it remains to be explored whether the beneficial effect of the co-treatment is mainly due to the blockade of the selected mediators.

Microglial Aβ clearance is critical for removing neurotoxic Aβ accumulated in AD brains. Our data show that the co-treatment increased Aβ clearance via PPARγ dependent pathway. Our data support the notion that PPARγ activation may be beneficial in AD due to its function in enhancing Aβ clearance. Of note, mice fed with a PPARγ ligand has been shown a reduction in Aβ deposition [41], however, it is not reported how the existing Aβ deposits are reduced. Based on our *in vitro* data of Aβ clearance, we speculate that the enhanced microglial Aβ clearance induced by PPARγ activation leads to reducing Aβ deposition in animals similar to what has been observed in Aβ immunotherapy studies, where microglial function was promoted by Aβ immunization (see [42] for a review). Clearance of Aβ requires Aβ uptake and Aβ degradation. Our data show that minimizing Aβ uptake by treatments of a PKC inhibitor did not affect the efficacy of the co-treatment on Aβ clearance, suggesting that Aβ degradation may play a major role in removing exogenous Aβ *in vitro*. Whether and how the co-treatment affects microglial phagocytic activity and/ or its degradation machinery remains to be investigated.

That Aβ-activated microglia-mediated neurotoxicity and microglial phagocytosis may be regulated differentially is supported by our previous studies and by others. Here, we showed once again that anti-inflammatory and Aβ clearance are promoted through different pathways by the co-treatment. Importantly, two favorable functions can be achieved pharmacologically by the co-treatment. Therefore, the co-treatment may have potential for AD therapy. The notion for PPARγ activation to be therapeutically useful in AD should be cautious because the blood-brain barrier permeability for15d-PGJ2 and other synthetic PPARγ ligands is poor. However, systemic administration of 15d-PGJ2 did improve the brain function through restoring the brain glucose and glutamate transporters in an animal model [43]. The possibility of the direct action of the drugs on the glail cells adjacent to the blood-brain barrier requires to be proved by further investigation.

In addition to revealing the multiple targeting properties of the co-treatment, our data showed that mechanisms underlying the beneficial effects of the co-treatment do not appear to be due to total deactivation of microglia. First, we found that the co-treatment did not reduce CD 11b immunoreactivity in primary cultures, as shown in Figure 3. Confocal images show that phagosome-like vehicles positive for CD 11b immunoreactivity became larger in cells with the co-treatment, suggesting an active state of microglia remains under circumstances of the co-treatment. Second, although the co-treatment did reduce the cell viability as measured by MTT assay, the co-treatment was able to promote microglial Aβ clearance *in vitro*. Thus, the beneficial effects of the co-treatment are unlikely due to elimination of activated microglia. Intriguingly, simultaneous treatment with the PPARγ antagonist did not improve cell viability, suggesting that pathways other than PPARγ activation are involved in the reduction of cell viability. Whether the co-treatment directly affects neuronal cell viability remains to be studied.

Pro-NGF was recently reported as a pro-apoptotic molecule in many models; its high affinity with p75NTR results in translocation of intracellular domain of p75NTR (p75ICD) to nuclei triggering cascades of apoptosis. The pro-apoptotic property of pro-NGF in the brain, however, remains obscure because pro-NGF is predominantly found in the brains of both normal and diseased. Since microglia are primary immune cells in the brain, altered secretion of the pro-apoptotic pro-NGF from microglia may contribute to microglia-mediated neuroinflammation. Thus, we speculate that microglial pro-NGF might play a role in AD pathogenesis during microglial activation. Surprisingly, we found that the Aβ/IFN-induced microglial activation, a model representing an acute neuroinflammation induced by stimulation of Aβ/IFN, showed a reduction of pro-NGF secretion by microglia, while LPS treatment lead to up-regulation of the secretion of pro-NGF. However, microglial activation did not lead to any changes in the intracellular level of pro-NGF. Since the level of microglial pro-NGF was not associated with the cell viability as discussed above, secretion of pro-NGF may be independent of the status of microglial activation and may not be necessarily detrimental. This notion is in agreement with findings by others demonstrating that two pro-NGF derivatives in additional to NGF may promote cell survival through Akt/PI3K [44]. Indeed, pro-NGF mediated p75NTR signaling in AD transgenic mice was not associated with increased neuronal death [45]. Furthermore, co-localization between microglial pro-NGF and CD11b in activated microglia as shown in Figure 3 implies that pro-NGF may be involved in the machinery of microglial phagocytosis toward Aβ. In fact, the association of p75NTR with phagocytosis has been reported in perivascular active cells [46]. The role of pro-NGF in CD11b-mediated phagocytosis in activated microglia remains to be explored and further studies are needed to illustrate the functions of microglial pro-NGF in AD.

## 3. Experimental

### 3.1. Animals and materials

For breeding, wild-type BALB/c mice were purchased from the National Laboratory Animal Center (Taiwan) and were maintained at the laboratory animal center of Taiwan’s National Health Research Institutes (NHRI). All procedures for animal handling were approved by NHRI’s Institutional Animal Care and Use Committee. Neonates (P1-3) were used for primary microglial culture as described below. PPARγ agonist, 15-d PGJ2, and RXR agonist, RA, were purchased from Sigma (St. Louis, MO, USA). PPARγ antagonist, GW9662, Protein kinase C inhibitor, BIM, and LPS were purchased from Calbiochem (Darmstadt, Germany). DP2 antagonist, BAY, and synthetic human Aβ1-42 were purchased from Cayman Chemical (Ann Arbor, MI, USA) and Bachem (Torrance, CA, USA), respectively. Antibodies for pro-NGF, 6E10, β-actin, CD11b, and MAP-2 were obtained from Alomone Labs (Jerusalem, Israel), Chemicon (Temecula, CA, USA), Abcam (Cambridge, UK), Serotec (Oxford, UK), and Lab Vision (Fremont, CA, USA), respectively. Antibodies for COX-2 and iNOS were obtained from BD Biosciences (San Diego, CA, USA). Mounting medium containing 4,6-diamidino-2-phenylindole (DAPI) was purchased from Vector Laboratories (Burlingame, CA, USA). Culture media, fetal bovine serum, and penicillin/ streptomycin were from Invitrogen (Carlsbad, CA, USA). Papain and DNase I were from Worthington Biochemical (Lakewood, NJ, USA). Enhanced chemiluminescence (ECL) was obtained from Amersham (Piscataway, NJ, USA).

### 3.2. Primary microglial cultures

Primary microglia cultures were derived from cortices of P1-3 neonates as described elsewhere [37]. Briefly, cells were dissociated using enzyme solution containing DMEM, EDTA (0.5 mM), L-cysteine (0.2 mg/mL), papain (15U/mL), Dnase I (200 µg/mL) followed by trituration and cultivated in culture medium containing DMEM supplemented with 10% FBS, 100 units/mL penicillin, and 100 µg/mL streptomycin. Microglia at the 14th day *in vitro* (DIV) were separated from the underlying astrocytic monolayer by gentle agitation using their differential adhesive properties. To determine purity of microglia, 1 × 10^4^ cells was cultured on chambered slide with treatment of 100 ng/mL of LPS for 24 h followed by cytochemistry analysis using a microglial marker, CD11b. Percent of positive microglia will be normalized by DAPI nuclei counterstaining. Purity of microglia was approximately 98%.

### 3.3. Aβ-induced immune activation

Aβ-mediated microglial activation was achieved by treating microglia with 15 μM of pre-aggregated Aβ1-42 and 10 ng/mL of IFNγ. Preparation of aggregated Aβ was made by incubation of 500 μM Aβ stock in PBS at 37 °C for 24 h as described previously [37]. In this preparation, various sizes of Aβ aggregates were observed by Western blot (data not shown). Microglia were seeded at 1 × 10^6^ in 6-well plates for 6 h followed by 1 h pre-treatment of drugs as detailed in Results. Pharmacological activation of PPARγ was performed by the co-treatment with the PPARγ natural ligand, 15d-PGJ2 (1 μM), and a RXR agonist, 9-cis-retenoic acid (10 nM). Immune activation was evaluated by expressions of cyclooxygenase-2 (COX-2) and inducible nitric oxide synthase (iNOS). Samples were subjected to electrophoresis and Western blot analyses using antibodies against COX-2 (1:750) and iNOS (1:750) followed by incubation of HRP labeled secondary antibodies. The corresponding bands revealed by ECL reaction were analyzed by Image J.

### 3.4. Aβ clearance assay

To evaluate the efficacy of Aβ removal, non-aggregated Aβ was applied for optimal microglial uptake [37] and the procedure was adapted from our previous work with some modifications [37]. Microglia were harvested and seeded at 3 × 10^5^ in 24-well plates for 6 h followed by 1 h pre-treatment of drugs as detailed in Results. Aβ was aggregated for 2 h at 37 °C in PBS and cells were incubated with Aβ at a concentration of 100 nM. After 24 h of incubation, culture medium were collected and subjected to brief centrifugation (300 × g at 4 °C for 10 min). The resultant supernatant was subjected to 16.5% Tris-Tricine gel electrophoresis and Western blot analysis for Aβ detection. Monoclonal antibody, 6E10, against Aβ was used. Aβ monomer at approximately 4 kDa was used for quantification because it was the detectable form in this system on Western blot. Aβ clearance was evaluated by measuring the corresponding bands visualized by ECL using Image J.

### 3.5. Pro-NGF measurement

Primary microglia were seeded at 3 × 10^5^ in 24-well plates for 6 h and cultivated in serum free DMEM with or without 1 h pretreatments as indicated in Results. Pre-aggregated Aβ (15μM) and IFNγ at 10 ng/mL were used to activate microglia. Some cells were treated for LPS at concentrations ranging from 0.001 to 10 μg/mL. Cultural media were subjected to evaluation for secretion of microglial pro-NGF using 10% Tris-HCl electrophoresis and Western blot analysis. Antibody against pro-NGF was used at 1:1000 for overnight incubation followed by incubation of HRP-labeled secondary antibody. The corresponding bands visualized by ECL were compared between treatments using Image J.

### 3.6. Cell viability assay

Primary microglia were seeded at 1 × 10^5^ in 96-well plates for 6 h and cultivated in serum free DMEM with or without 1 h pretreatments as indicated in Results. Cell viability was evaluated using MTT (3-(4,5-dimethylthiazol-2-yl)-2,5-diphenyltetrazolium bromide) assay per the manufacturer’s directions (Invitrogen, Carlsbad, CA, USA). Results were obtained using a Multiskan EX plate reader (Thermo Electron, Waltham, MA, USA).

### 3.7. Statistics

A two-tailed independent *t*-test was used to test the significance. Significance for Post Hoc multiple comparisons between treatments was adjusted by Bonferroni using SPSS software. Data are presented as means ± SD.

## 4. Conclusions 

We found that co-treatment of RA and 15-d PGJ2 was able to manipulate microglial activation by alleviating Aβ/IFNγ-induced immune activation and by enhancing microglial Aβ clearance simultaneously, which is a favorable combination in AD therapy. Our data support the notion that the co-treatment of RA and 15-d PGJ2 may be beneficial for AD therapy. Surprisingly, there was no clear association between level of secreted pro-NGF from microglia and the Aβ/IFNγ-activated microglial activation. Our data imply that the proposed pro-apoptotic property of pro-NGF may not contribute to the Aβ/IFNγ-activated microglia-mediated neuroinflammation. That Aβ/IFNγ reduced secretion of microglial pro-NGF may suggest an alternative role of A in AD pathogenesis. 

## Figures and Tables

**Table 1:** Co-treatment of RA and 15d-PGJ2 suppressed Aβ/IFNγ-induced immune activation in primary microglia. Data were presented as % of the expression level in the Aβ/IFNγ-treated cells.

	iNOS	COX-2
Aβ/IFNγ + RA/15d-PGJ2	55.4 ± 30.3%*, *n* = 5	47.2 ± 19.7%**, *n* = 5
Aβ/IFNγ + RA/15d-PGJ2 + GW9662	51.4 ± 27.3%*, *n* = 5	60.3 ± 24.7%**, *n* = 5
Aβ/IFNγ + RA/15d-PGJ2 + BAY	56.6 ± 29.1%*, *n* = 6	48.8 ± 19.3%**, *n* = 6
Aβ/IFNγ + RA/15d-PGJ2 + BIM	44.0 ± 9.5%**, *n* = 4	46.4 ± 12.0%**, *n* = 4

Significance for comparisons with the controls is indicated as *p < 0.05, **p < 0.01.
